# The Impact of Orthodontic Treatment on Masticatory Performance: A Literature Review

**DOI:** 10.7759/cureus.30453

**Published:** 2022-10-19

**Authors:** Giovanna Zanon, Luca Contardo, Bachar Reda

**Affiliations:** 1 Department of Medical, Surgical and Health Sciences, University of Trieste, Trieste, ITA

**Keywords:** swallowing, orthodontic therapy, orthodontics, masticatory performance, chewing

## Abstract

The aim of this narrative review was to evaluate the possible association between orthodontic therapy and improved masticatory function. A search strategy was conducted using the PubMed database for up to January 2020 using the keywords “mastication” and “orthodontics”. Only human studies investigating mastication in orthodontics settings were selected. The search strategy resulted in 1,011 articles, out of which 57 were included in the final analysis. Investigations have generally agreed that masticatory and chewing functions improved post-orthodontic and orthognathic treatments. Studies also showed improvement in the quality of life of patients' post-orthodontic treatment. The articles examined reinforced that besides esthetic reasons, orthodontic therapy does improve the masticatory and chewing functions of individuals, ultimately enhancing their health-related quality of life.

## Introduction and background

Mastication is considered the first stage of the digestive process, whereby the food consumed is broken down into smaller particles. The structural changes that occur to the food before swallowing differ according to its physical state. Liquid foods do not undergo any structural changes, whereas solid foods are chewed and broken down before swallowing. Malocclusion can negatively impact individuals’ ability to process and break down food [1]. Understanding the mechanisms involved in the process of mastication and swallowing is therefore important to provide a therapy tailored to the esthetic and functional needs of patients [2].

Orthodontics is that branch of dentistry concerned with facial growth, with development of dentition and occlusion, and with the diagnosis, interception, and treatment of occlusal anomalies [3]. Restoring physiologically normal masticatory function post-orthodontic treatment is a vital goal that should not be overlooked [3]. Studies have shown that orthodontic treatment also improves occlusal force and occlusal contacts [4]. The medical literature has also seen an increasing emphasis on the impact of orthodontic treatment on improving health-related quality of life [5]. The aim of this narrative review was to assess the direct evidence regarding the effect of orthodontic therapy on the masticatory and chewing functions.

## Review

Materials and methods

For this narrative review, a literature search was conducted using the PubMed database for up to January 2020 using the keywords “mastication” and “orthodontics”. No limits were set on the year of publication, and only studies published in the English language were considered. The studies were heterogeneous. Randomized controlled trials, longitudinal prospective studies, case report articles, cohort studies, case-control studies, prospective studies, review articles, systematic reviews, and observational studies were eligible for inclusion. All titles and abstracts were screened by the authors (GZ, LC, BR), and only studies that considered both orthodontics and mastication have been carefully scrutinized. The reference lists of main studies were manually searched to make sure no relevant publication was missed in the database search.

Results

The database search yielded 1,011 potential articles. After evaluation of the titles and abstracts, articles that did not correlate mastication with orthodontics, articles that were not in English, and animal studies were excluded; eventually, 57 articles were selected, fully scrutinized, and included in the review. No additional articles were identified after searching the reference lists of the initially selected articles.

Five of the selected studies focused on the perceived and actual need for orthodontic treatment [6-9] and assessed patients’ satisfaction at the end of the orthodontic-surgical treatment [10]. Fifteen studies investigated the masticatory and chewing functions of patients before and after orthodontic treatment [4,11-24]. Two studies assessed the change in occlusion and masticatory function post-orthodontic treatment and evaluated the self-perceived masticatory function with tested masticatory efficiency among both treated and untreated groups [25,26]. Thirteen studies evaluated the impact of orthodontic appliances and therapies on masticatory and swallowing functions [1,27], perceived and actual pain sensation [28-30], and other related aspects such as oral comfort, speech, and dietary habits [27,31-38]. Two studies evaluated the masticatory performance and temporomandibular disorders (TMDs) in patients before and after orthodontic and orthognathic treatments [39] and the improvement of the masticatory movement in patients after orthognathic treatment [40]. Four studies presented different treatment options for malocclusions and examined the impact of masticatory performance on patients’ quality of life [3,24,41,42]. Three studies investigated the improvement of masticatory performance following orthodontic treatment [43-45]. Five studies evaluated the masticatory muscle performance before, during, and after orthodontic [46-48] and orthognathic treatments [49,50]. Two studies investigated the effect of orthodontic appliances and mastication on friction and the risk factors that predict composite attachment loss in patients [51,52]. Two studies [53,54] focused on the use of lingual appliances and oral disturbances associated with their use. Three studies [55-57] emphasized including orthodontic therapy in the multidisciplinary treatment of isolated cleft lip or unilateral cleft lip and palate (UCLP). All these studies indicate the effect of orthodontic treatments in improving mastication. These studies also implicate that orthodontics is much more complex and requires a multidisciplinary approach to identify the causes impacting masticatory performance in order to identify the proper treatment.

Discussion

Difference between the perceived and actual need for orthodontic treatment

Studies investigating the extent to which adults [7,9] and adolescents [6,8] perceive the need to undergo orthodontic treatment and the extent to which this perception reflects the actual reality of the situation showed that the younger generation has been esthetically more concerned with their oral health and is giving greater attention to the condition of their occlusion. Cultural influence has been reported to be the main force of such a decision [6], followed by the need to improve chewing performance, with the latter being valued mainly post-treatment [10,58].

Difference in masticatory and chewing functions pre- and post-orthodontic treatment

Fourteen studies [4,11-23] investigated the changes in masticatory and swallowing patterns that occurred before and after orthodontic treatment. Studies also showed that correcting malocclusions can improve symmetry in chewing movements [12-14,19,23], improve masticatory function [11,14-18,20,22], improve food break-down into smaller pieces [4], and prevent deviation of the mandible during opening movements [17].

Subjective perception of masticatory capacity measured in treated and untreated groups

Studies examining patients’ perceptions of changes in masticatory functions following orthodontic treatment revealed significant patient awareness of the improvements that occur throughout the therapy. Nevertheless, it is suggested that patients in class I have better masticatory function than those with malocclusions [25,26].

Impact of orthodontic devices and therapy on masticatory function and pain

Studies assessing the impact of using orthodontic devices on masticatory function [1,27,32] and pain [28,29] showed that pain due to orthodontic devices during orthodontic treatment was a major obstacle to encouraging patients to undergo orthodontic treatment. This pain, however, was solely felt when applying the devices. So, patients were required to restrict their diet, whereby only soft food choices were consumed, rarely resorting to analgesics [30,31,33-38].

Relationship between temporomandibular disorders (TMDs) occurrence and masticatory function before and after orthognathic treatment

Although current evidence fails to show a causative relationship between malocclusion, orthodontic treatment, and temporomandibular disorders, some reports still suggest that patients with dental and facial deformities are more likely to suffer from temporomandibular disorders (TMDs). Orthodontic therapy in combination with orthognathic surgery may or may not have an influence on the occurrence of symptoms. It has been seen that subjects undergoing such treatments may experience less myofascial pain and arthralgia [39,40]. Other studies, however, have shown that the symptoms of pre-surgery TMDs have an uncertain course probably unrelated to the surgery itself [41]; other studies suggested that orthognathic surgery has no effect on the said symptoms [42,43]. Finally, some studies have concluded that counterclockwise rotation of the jaw in class II patients increases TMD-related symptoms [44,45].

Changes in dietary habits with the use of orthodontic devices

Patients reported changes in their dietary habits during the first few days of treatment. Pain, difficulty in biting [38], and fear of breaking the device [27] were reported to be the main predictors of this change. Nutritional and dietary consultation should be provided before initiating the treatment.

Treatment of malocclusions

Orthodontic therapy techniques vary according to the patient’s case. For worst-case scenario, orthognathic surgery is considered [3,24,46,47]. Studies showed that with proper therapy, masticatory and swallowing functions are more likely to improve [3,24,46,47].

The need for orthodontic treatment

Patients requiring orthodontic treatment experience significant improvement in the masticatory and chewing functions post-therapy. Studies had shown that such an improvement had been subjectively perceived by patients, whereby an increase in patient satisfaction has been reported post-treatment [48-50].

Improvements in masticatory muscles during orthodontic treatment

Orthodontic treatments positively impact the masticatory system [51]. Studies have demonstrated that orthodontic treatment regulates muscular activity by training muscles to function symmetrically. It has also been suggested that orthodontic treatments positively benefit the temporomandibular joints [52]. In fact, some studies have shown a decrease in the masseter contraction after orthodontic correction [53,54]. In conclusion, orthodontic therapy eases and regulates muscular activity, as well as regulates masticatory and chewing functions [55].

Improvement in mastication following orthodontic therapy

Studies have suggested that the masticatory function varies relative to different malocclusions. Throughout the therapy, improvements are noticed particularly in areas with greater malocclusions [18]. Orthodontic appliances were also proven to lower the percentage of reverse-sequencing chewing cycles by improving their kinematic parameters (axis, closure angle, maximum lateral excursion) on both sides, therefore becoming symmetric between sides [22]. It has also been reported that patients were able to detect improvement in their masticatory function throughout the therapy [56].

Factors that impact the effectiveness of the orthodontic appliances

There are several factors that impact the effectiveness of orthodontic devices used during the treatment [57,58]. Studies have shown that chewing does not have a significant effect on the effectiveness of orthodontic devices over time but rather on the intraoral environment [57]. Factors such as one-sided mastication and frequent removal of the aligner have been mainly associated with damaging the devices, which could over time negatively affect the outcomes [58].

The use of lingual devices

There is little evidence regarding [59,60] the use of lingual braces in the treatment of malocclusion and their impact on masticatory and chewing functions. Although lingual devices have been widely used due to their esthetic considerations, evidence remains insufficient to validate any results [60].

Orthodontic treatment in patients with cleft lip and unilateral cleft palate (UCLP)

Studies suggested that orthodontic treatment is important to improve the masticatory function of patients with unilateral cleft lip and palate (UCLP) [61] and cleft lip [62], although considered less ideal as compared to regular patients [63].

## Conclusions

After careful analysis of the studies found in the literature, we may conclude that orthodontic therapy has a major impact on the masticatory and chewing functions of individuals. Additionally, it seems that orthodontic treatments can improve the quality of life of patients post-treatment. Nevertheless, pain during treatment remains a major barrier for many patients. Providing patients with dietary guidelines prior to the initiation of treatment appears crucial to preserve the appliances and avoid discomfort during treatment. Uncertainties remain in relatively new areas (such as the use of lingual devices); however, this subject area is more concerned with the esthetic aspect rather than the physiological one.
